# Strategies for implementing pet robots in care homes and nursing homes for residents with dementia: protocol for a modified Delphi study

**DOI:** 10.1186/s43058-022-00308-z

**Published:** 2022-06-03

**Authors:** Wei Qi Koh, Dympna Casey, Viktoria Hoel, Elaine Toomey

**Affiliations:** 1 College of Medicine, Nursing and Health Sciences, National University of Ireland Galway, H91 E3YV Galway, Ireland; 2 Institute for Public Health and Nursing Research, University of Bremen, 28359 Bremen, Germany; 3 Leibniz Science Campus Digital Public Health, 28359 Bremen, Germany; 4 School of Allied Health, University of Limerick, V94 T9PX Limerick, Ireland; 5 Health Research Institute, University of Limerick, V94 T9PX Limerick, Ireland

**Keywords:** Implementation strategy mapping, Implementation strategies, Pet robots, Social robots, Implementation, Care homes, Nursing homes, Consensus study, Dementia

## Abstract

**Background:**

Pet robots are a type of technology-based innovation that have shown positive psychosocial benefits for people with dementia in residential facilities, such as improving mood and social interaction and reducing agitation. Nevertheless, little is known about how pet robots can be implemented in care homes and nursing homes for dementia care in real-world practice. The objectives of this study are to (1) identify contextualised implementation strategies for implementing pet robots into care homes and nursing homes for dementia care and (2) achieve consensus on the most relevant strategies.

**Method:**

This study is informed by a preceding scoping review and qualitative study, which used the Consolidated Framework of Implementation Research (CFIR) to identify multi-level determinants of implementation (i.e. barriers and facilitators). We will use the CFIR-ERIC matching tool to identify relevant implementation strategies from the Expert Recommendations for Implementing Change (ERIC) taxonomy to address these determinants. Data from the scoping review and qualitative study will be used to contextualise the generic ERIC strategies for our setting. After that, a group of key stakeholders will be consulted to further contextualise and refine these strategies. Next, a two-round modified Delphi process will be conducted. Fifty-four international expert participants including healthcare professionals and organisational leaders from care homes and nursing homes and academic researchers will be recruited through purposive sampling. During the first Delphi round, participants will be invited to rate the relevance of each implementation strategy on a 9-point Likert scale and provide comments or suggestions. Descriptive statistics will be used to identify whether consensus has been obtained. Inductive qualitative content analysis will be used to analyse and summarise textual responses for any new statements suggested by participants. Statements that do not reach consensus and new statements suggested in round 1 will be taken to the next round, which will follow the same rating process.

**Discussion:**

This study will identify strategies for implementing pet robots in care homes and nursing homes for residents with dementia, which will have practical utility for clinicians, organisations and researchers. It will also demonstrate the practical application (and adaptation) of the CFIR-ERIC tool to identify and contextualise ERIC strategies.

**Supplementary Information:**

The online version contains supplementary material available at 10.1186/s43058-022-00308-z.

Contributions to the literature

- Pet robots are technological innovations to benefit the psychosocial health of people with dementia. However, little is known about how they can be implemented in real-world practice in care homes and nursing homes
- This study will use expert consensus to identify the most relevant strategies for guiding the implementation of pet robots in care homes and nursing homes for dementia care
- This study will demonstrate the practical application of theory, using the ERIC taxonomy of implementation strategies and the CFIR-ERIC tool, to guide the identification and systematic contextualisation of implementation strategies using empirical data

## Introduction

Pet therapy, or animal-assisted therapy, has shown positive psychosocial benefits for people living with dementia (PLWD), such as improving mood and social interaction and reducing agitation [1]. Nevertheless, the use of live animals can pose practical and logistical challenges, such as the potential transmission of zoonotic diseases, or cause unintended injury to the animal or to the person living with dementia [1]. Since the early 2000s, pet robots have emerged as technology-based substitutes for pet therapy. Early examples include Aibo, a robotic dog encased in a plastic shell, and PARO, a realistically designed baby harp seal robot covered in a soft fur coat. PARO was developed to support the social and emotional needs of older people, including people with dementia. In the last decade, developers have continued to develop pet robots to encompass different design features. Examples include Pleo, a robot dinosaur; CuDDler, a robot bear; and the Joy for All (JfA) cat. Studies have shown that older adults and PLWD prefer realistically designed pet robots that are covered in soft fur coats and have cited the JfA cat as their preferred design [2]. Numerous empirical studies have been conducted to investigate the effectiveness and impacts of pet robots for PLWD in long-term residential care, such as care homes and nursing homes [3–7]. Synthesised findings suggest that the use of pet robots for PLWD resulted in reduced behavioural and psychological symptoms of dementia (BPSD), reduced agitation, improved mood and improved social engagement [3–5]. Whilst most effects have not been statistically significant due to small sample sizes and intervention heterogeneity, pet robots show promise as non-pharmacological solutions to improve the psychosocial health of PLWD [3–5]. Despite numerous studies that have been conducted on their effectiveness and demonstrated their promise, the uptake of pet robots in real-world practice remains low [8–10]. This is because traditionally, research follows a stepwise process, where the efficacy and effectiveness of an intervention have to be confirmed before its implementation is investigated [11, 12]. However, this stepwise approach to research has promulgated a marked time lag between research discovery and uptake in real-world practice [11, 12]. In other words, to improve the speed of knowledge creation and to improve the clinical relevance of pet robots in real-world practice, it is important to pursue knowledge on their implementation alongside investigation into their effectiveness [13, 14].

### Determinants of implementation

To move pet robots into routine dementia care practice in care homes and nursing homes, it is important to first understand the determinants (i.e. barriers and facilitators) influencing their implementation. In a recent scoping review, we explored barriers and facilitators that influenced the implementation of social robots, including pet robots, for older adults and PLWD [4]. The findings were synthesised using the Consolidated Framework of Implementation Research (CFIR), a framework that has guided the comprehensive exploration of implementation determinants. Within the CFIR, 39 constructs are grouped into five domains: (1) intervention characteristics, (2) outer setting (i.e. external influences on the implementing organisation), (3) inner setting (influences within the implementing organisation), (4) characteristics of individuals involved in the implementation and (5) implementation process. Barriers and facilitators from 53 included studies were mapped onto 18 CFIR constructs across five domains. The findings showed that existing studies have been largely focused on investigating the internal validity of social robots, and there has been a scarcity of studies that investigated contextual factors relating to their external validity. Consequently, we conducted a qualitative study, guided by the CFIR, to address gaps that were identified in the scoping review and to further understand the barriers and facilitators to the implementation of pet robots in nursing homes for PLWD [15, 16]. Barriers included a lack of customisability to suit residents’ abilities and preferences, doubts about long-term use, prohibitive costs, lack of external funding, resources and knowledge, infection prevention mandates and conflicting stakeholder views on the anthropomorphisation of pet robots [16]. Facilitators included the realisticness and familiarity of pet robots, identification of residents’ needs that can be met or were met using a pet robot, compatibility with prevailing regulatory guidelines and organisational care processes, intrinsic desires to improve residents’ quality of life and buy-in from stakeholders [16].

### Implementation strategies

Following the identification of implementation determinants, implementation strategies that are feasible, effective and contextually relevant that specifically target those determinants need to be identified to guide their implementation in practice. Implementation strategies are defined as “methods or the techniques used to enhance the adoption, implementation and sustainability of a clinical programme or practice” [17]. They can include single methods (i.e. discrete strategies) or a combination of methods (i.e. multifaceted strategies) that are chosen to enhance the implementation of an intervention. Powell and colleagues developed the Expert Recommendations for Implementing Change (ERIC), a taxonomy of 73 implementation strategies based on a review of implementation taxonomies, reviews and compilations, conceptual papers and empirical papers [18], and has been previously validated through a modified Delphi process involving clinicians and implementation scientists [19].

### Mapping determinants to implementation strategies

There is little guidance in the implementation science literature about how to systematically select strategies to address implementation determinants [20]. Therefore, in practice, the selection of strategies does not always follow the determinants identified [20]. To address this, Waltz and colleagues developed the CFIR-ERIC mapping tool, which was intended to map the barriers that have been coded to CFIR constructs onto ERIC implementation strategies [21]. However, as the CFIR constructs are often considered as determinants (i.e. barriers or facilitators), both identified barriers and facilitators may be mapped onto the tool, to generate potentially relevant strategies to address CFIR-coded barriers and strengthen CFIR-coded facilitators [22, 23]. The outputs of this tool include implementation strategies in relation to the input on CFIR determinants, along with percentages, which reflect the proportion of experts that have endorsed the strategy as being appropriate to address each CFIR determinant [21]. It has been previously used in empirical studies to guide the identification of implementation strategies [24–26], (www.cfirguide.org/choosing-strategies). This study aims to use our previous studies [15, 27] to identify relevant implementation strategies for identified implementation determinants, contextualise the strategies for our setting and obtain expert consensus on the most relevant strategies for implementing pet robots in care homes and nursing homes for PLWD.

## Objective

The objectives of this study are to:

1. Identify and contextualise the strategies for implementing pet robots into care homes and nursing homes for dementia care
2. Achieve consensus from a panel of international experts on the most relevant strategies for implementing pet robots in care homes and nursing homes for PLWD.

## Method

### The Delphi technique

The Delphi technique is a research method that allows for the structuring of group communication, through a multistage process of sequential surveys or rounds [28], to allow “a group of individuals as a whole to deal with a complex problem” [29]. It is used where the judgement of individuals (experts) can be combined to address a knowledge gap or lack of agreement [29]. The modified Delphi is a variant of the classical Delphi [30], where the first qualitative round is omitted when statements for the survey can be derived from literature or previous research [31]. This has been recommended for use (in place of the classical Delphi) to enhance study validity, since using an initial qualitative round to generate statements can subject the initial statements to biases [32, 33]. For instance, the number of experts and their levels of expertise can influence the validity of the statements. Furthermore, initial qualitative responses that are gathered may create ambiguous and generic statements, which could lead to biases at the outset [32, 34]. As such, using the modified Delphi technique can enhance the content and face validity of the survey [32].

**Fig. 1:**
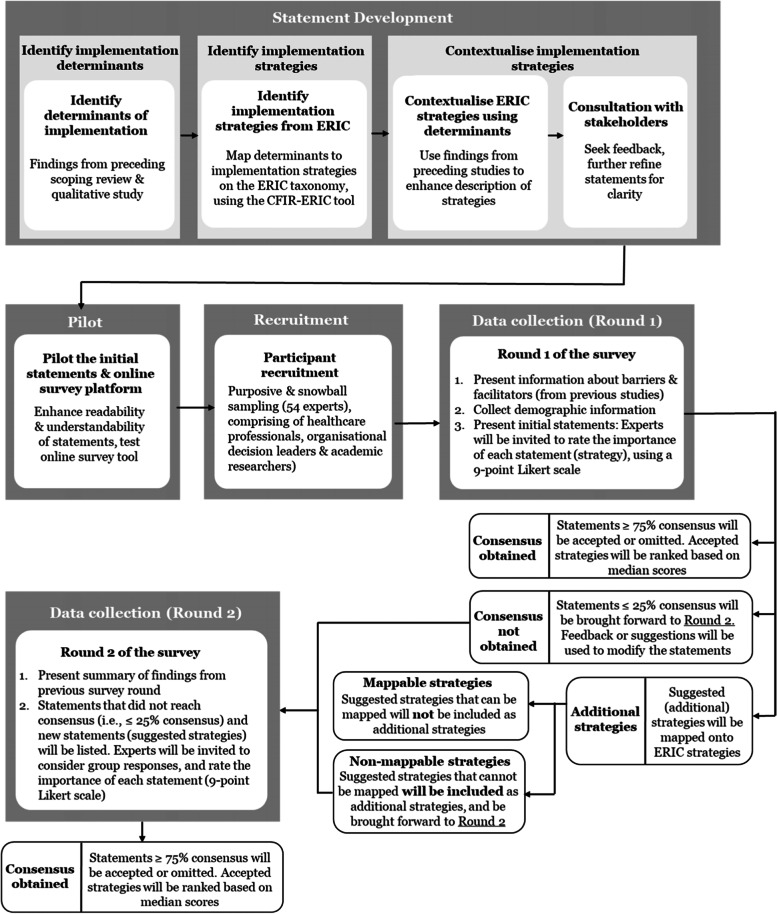
Flowchart of the study process

A two-round modified Delphi process was chosen as the most appropriate research method to address the research objective, as findings from the preceding qualitative study will inform the initial statements for the first survey round. The Conducting and REporting DELphi Studies (CREDES) guidelines [35] will be used to guide the design, conduct and reporting for this study (Additional file 1). An overview of the study process can be found in Fig. 1.

### Statement development

The determinants of implementation identified from our preceding scoping review [27] and qualitative study [15] and coded to CFIR will be used as a starting point. We will identify ERIC implementation strategies relevant to these determinants using the CFIR-ERIC mapping tool. This will allow a list of potentially relevant implementation strategies to be generated, along with a cumulative percentage to indicate the collective relevance of each strategy in addressing CFIR determinants. Whilst all the strategies in the ERIC taxonomy may be potentially relevant, not all should be considered as Delphi statements, as a longer list of statements has been associated with significantly lower response rates [29]. To strategically balance the number of prospective statements with overall comprehensiveness, only implementation strategies with a cumulative percentage of over 100% will be selected.

The definitions (descriptions) of the ERIC implementation strategies are generic by design, as the authors intended for them to be broadly applicable [29]. Accordingly, we will contextualise them for our setting by using the data from our preceding scoping review and qualitative study. The determinants identified in these studies will be used to describe each ERIC strategy. To minimise subjectivity in this process, this exercise will be verified by a second researcher. Meetings will be held to discuss any disagreements, until consensus has been met. Finally, to further contextualise the implementation strategies, we will purposively identify and consult with key stakeholders to discuss and refine the clarity and definition of each strategy. This will include at least one healthcare professional, one organisational leader from a care home/nursing home, one academic researcher and one Patient and Public Involvement (PPI) member from the Dementia Research Advisory Team [36]. These stakeholders will not be involved in the actual Delphi process. The identification and recruitment of these stakeholders will follow the same process as participant recruitment (outlined in the later section on recruitment).

Finally, the statements and the Delphi process itself will be piloted to further refine and enhance the clarity of the statements and to address any potential issues related to the online survey platform [37]. The final statements will constitute the initial statements for the first Delphi round.

### Participants (expert panel)

Baker and colleagues (2006) suggested that the knowledge and experience of individuals should be taken into account. Topic area knowledge relates to an individual’s professional and/or academic qualification, which demonstrates that he or she has a level of predefined knowledge base in a topic or clinical area. The authors’ and co-authors’ peer-reviewed publications are often considered as knowledge experts [38]. Experience-based expertise refers to an individual’s level of clinical or practical experience in relation to the research topic [38]. As it is not possible to ascertain expertise solely based on the length of time spent in a field [38], another experience-based criterion such as the nature of the individual’s experience should also be considered. Whilst some authors have argued for a homogenous sample of participants for Delphi studies [39], others assert the need for a heterogeneous sample [40–42] to increase validity through incorporating diverse and varied perspectives [43]. Based on these considerations, participants for this Delphi study will be selected for (1) topic-based knowledge expertise, as demonstrated through academic publications in relevant topic areas, and (2) experience-based expertise, as demonstrated through practical experience. These will be outlined in the inclusion criteria below. The three main groups of participants will include (i) healthcare professionals with experience in providing dementia care in care homes/nursing homes, (ii) organisational decision-makers from care homes/nursing homes and (iii) academic researchers. Although PLWD are service users of pet robots, we did not include them as participants due to our focus on healthcare provider and organisational related contexts. For clarity purposes, in this study, care homes and nursing homes are defined as institutions or facilities that provide long-term residential care and support and/or nursing care for residents [44]. To support the external validity of the study findings, we aim to recruit participants from within Ireland and internationally.

#### Care professionals

Care professionals such as nurses, healthcare assistants activity coordinators and allied health professionals (e.g. occupational therapists, physiotherapists and therapy assistants) can provide experience-based expertise about the implementation of pet robots for PLWD in care homes and nursing homes, as they influence the process of direct care provision. Healthcare professionals who meet the following criteria will be included:

- Have current or previous experience of providing care to PLWD in a care home or nursing home(s)
- Can read and understand English

#### Organisational decision-makers (ODM)

Organisational decision-makers, such as team leaders, managers and directors, may be considered as indirect care providers who provide care services that may not require interaction between the provider and the PLWD. It is necessary to involve this group of key stakeholders, as they can offer experience-based perspectives on the implementation strategies from a managerial point of view. Organisational decision-makers who meet the following criteria will be included:

- Have current or previous experience as a manager/leader in a care home or nursing home that provides care for PLWD
- Can read and understand English

#### Academic researchers

Academic researchers with publications in the field of implementation science and in using technology and/or psychosocial interventions in dementia care in care homes or nursing homes can contribute valuable topic-based knowledge expertise. As such, they are an important stakeholder group that should be included in the expert panel. Academic researchers who meet the following criteria will be included:

- First, second or last author in at least one peer-reviewed publication in at least one of the following research fields within the last 10 years: (i) implementation research in care/nursing home settings, (ii) psychosocial interventions for PLWD in care/nursing homes or (iii) using technology for dementia in care/nursing homes
- Can read and understand English

### Recruitment

#### Healthcare professionals and organisational decision-makers

First, the lead researcher (WQK) will contact care homes/nursing homes in Ireland and in the United Kingdom (UK) that provide care for PLWD. Based on the list of homes identified on the Irish open data portal [45], the researcher will systematically identify ones that provide care for PLWD, using information from the Health Information and Quality Authority (HIQA) inspection reports. Organisations will be informed about this study and invited to disseminate information about this study to the staff. Care homes and nursing homes in the UK will be identified in collaboration with the Enabling Research in Care Homes (ENRICH), using the same process as outlined above. Second, the researcher will advertise the study through social media and by reaching out to healthcare professional bodies in the UK and Ireland, such as the Association of Occupational Therapists of Ireland and the National Activity Providers Association (UK), who will be invited to disseminate notices of this study to members. Those who express interest will be invited to contact the researcher for more information. Finally, the researcher will draw on her networks and connections to identify prospective participants.

#### Academic researchers

The first, second and/or last authors in peer-reviewed publications in implementation research, using technology and/or psychosocial interventions in dementia care in care homes/nursing homes, will be identified and invited to participate. Next, an email will be sent to a representative from the INTERDEM (early detection and timely INTERventions in DEMentia) network, a pan-European network of dementia researchers, who will be invited to disseminate information on this study to researchers in the network. Notices of this study will also be advertised through social media. Lastly, the researcher will also draw on her networks and connections, such as the Dementia: Intersectoral Strategy for Training and Innovation Network for Current Technology (DISTINCT) consortium. Similarly, those who express interest will be invited to contact the researcher for more information and invited to participate if eligible.

### Sampling strategy

Purposeful sampling will be used to select experts for the study based on the expertise and experiences of individuals [42], as per the inclusion criteria. Snowball sampling will also be used as a secondary sampling technique. Participants will be asked if they have colleagues who would be eligible and interested, who can contact the researcher to discuss participation.

### Sample size

There is no set standard for a sample size of a modified Delphi panel. It has been suggested that the number of panellists could range from 10 to 18 panel members per area of expertise [46–48]. Taking into consideration the median sample size based on these recommendations for a total of three groups of key stakeholders, the target sample size for this study is 42 participants (i.e. 14 panel members per area of expertise). Because the Delphi technique requires time and participants’ commitment, a dropout is likely to happen [49, 50]. Retention rates throughout the Delphi process from the first to the final round have not been reported consistently in the literature [50] and have ranged from 19.5 to 87.1% [37, 51–54]. In consideration of the lower attrition margin of 20%, an initial sample of 54 participants will be recruited (i.e. 18 participants from each group). Measures will be taken to maximise the retention of participants, and this will be described in the later section.

### Data collection and analysis

Data collection is expected to start in March 2022 and is expected to be completed within a 3-month period by May 2022. Information about the implementation determinants will be provided to participants, and two rounds of the survey will be administered via an online platform (QuestionPro) and distributed to individual participants via email.

#### Round 1

The first round of the survey will include three sections. In the first section, an executive summary of the determinants of implementing pet robots, based on the barriers and facilitators identified from preceding studies, will be presented. This is an important step for participants to have knowledge on the identified determinants of implementing pet robots before commencing the survey to identify relevant strategies. In the second section, demographic information will be collected. This will include information such as participants’ gender and profession. Information about expertise will also be collected. For academic researchers, information about the number of years working in the field of implementation science and/or dementia research will be collected. For healthcare professionals and organisational decision-makers, respective information about the number of years providing care for PLWD and the number of years that they have held a management role in care home(s) or nursing home(s) will be collected.

In the third section, participants will be presented with statements and be invited to rate the importance of the implementation strategies. Whilst there is no gold standard for selecting an appropriate scale for consensus processes to identify implementation strategies [55], 9-point scales have been recommended by the Grading of Recommendations Assessment, Development and Evaluation (GRADE) Working Group to assess the importance of research evidence [56] and have been suggested to have more discriminatory power than other scales [57]. As such, a 9-point Likert scale will be used. A score of 1 to 3 indicates limited importance; 4 to 6 indicates that a strategy is important but not critical; 7 to 9 indicates that it is important and critical. A comment box will also be included for each statement, where participants will be invited to provide optional explanations for their responses and/or offer suggestions to revise its definition. At the end of the survey, participants will be given the option to suggest up to three additional strategies that they feel warrant inclusion. The survey will be fully anonymised to ensure that dominant participants do not unduly influence group consensus. Participants will be given up to 3 weeks to provide responses for each modified Delphi round. Email reminders will be sent at weekly intervals.

Data generated from the first round will be extracted for analysis on the Statistical Package for the Social Sciences (SPSS) version 21. Descriptive statistics will be used to identify whether consensus has been obtained. Inductive qualitative content analysis will be used to analyse and summarise textual responses [58]. The distribution of participants’ responses, including the median and interquartile range of responses [59], will be calculated to determine the level of consensus and the extent (ranked based on the median scores) to which experts found an implementation strategy important. Based on the results, statements that achieved 75% consensus (at least 44 out of 54 participants rating a statement with a score of 1 to 3, 4 to 6, or 7 to 9) will be accepted or omitted from the recommendations. This level of agreement is based on the findings from a systematic review, which found that 75% consensus was deemed the most appropriate cut-off point in previous Delphi studies [60].

The Kruskal–Wallis test will be used to test whether the groups of experts differed significantly from each other in opinion about the implementation strategies. Items on which the groups differed will be further explored using the post hoc pairwise Mann–Whitney *U* test, to investigate which groups differed from each other. Statements not meeting the 75% agreement will be brought forward to the next round. If feedback or suggestions are provided, they will be used to modify the statements. Additional strategies that are suggested by participants will be mapped onto the list of ERIC strategies—if the suggested strategy has already been included as an ERIC strategy, it will not be included as a new strategy. Conversely, if they have not been included as an ERIC strategy, it will be listed as a new strategy and brought forward to the next survey round.

#### Round 2

The second round of the survey will explore if further consensus can be reached for items for which there was no consensus obtained in the first round. In the first section, findings from the previous round will be presented. This includes the list of statements that did not meet consensus and feedback of statistical data and comments to allow them the opportunity to reflect on the group response and reconsider their initial responses [61]. In the second section, statements that did not meet consensus and new statements (suggested additional strategies) will be listed. Like the previous round, participants will be invited to rate the relevance of the statements on the 9-point Likert scale. They will be given up to 3 weeks to provide responses. Similar to the previous round, email reminders to complete the survey will be sent weekly. Data analysis will follow the same process as described in round 1. Statements will be included in or omitted from the list of recommended implementation strategies if a consensus of 75% has been achieved, ranked and reported according to median ratings.

### Participant retention

To minimise attrition rates, it is important to keep participants fully engaged in the study [62]. Different methods will be used to maximise the retention of participants. First, engagement barriers related to comprehension [37] will be minimised through the process of consulting with stakeholders to contextualise statements and by piloting the statements. Second, participants will also be provided with explicit expectations about the intended time commitments and tasks, which includes clear information at the outset of the study to ensure that each participant will know how much time they will be expected to contribute (including the expected duration of the study), what they will be asked to do [37, 63]. Third, individualised emails will be used to remind and encourage participants to complete each round of the survey [37]. In the emails, the researcher will also emphasise that their expertise and views were important and provide an update on the number of experts that have completed the survey so far [37]. These strategies have been reported to be helpful for maximising participant retention [37, 64, 65].

## Rigour

Several strategies will be taken to ensure rigour, so that the use of the Delphi technique can be considered a reliable and credible source of evidence. First, instead of using a classical Delphi (where a qualitative first round is used to generate statements), statements for this study will be generated from the findings of two preceding research studies. A structured process and stakeholder consultation will be used to guide the selection and refinement of implementation strategies—this arguably enhances the reliability [66], content validity and face validity of the initial statements [32]. Next, construct validity will be ensured through the process of Delphi iterations. As the researcher summarises group responses from each Delphi round and shares the summary with experts, it provides them with the opportunity to check and validate their responses [48, 67]. Finally, the CREDES guidelines will be used to enhance rigour during the conduct of the study and guide the transparent reporting of the findings [35].

### Limitations

The potential limitations of this study should be acknowledged. First, our preceding qualitative study (to explore implementation determinants) was conducted with participants from nursing homes in Ireland, which may limit the generalisability of these determinants. Nevertheless, these findings were triangulated with findings from our scoping review, which synthesised findings from studies conducted in other countries. Next, whilst several data sources and expert opinions were sought to develop the ERIC taxonomy and CFIR-ERIC tool, which can guide the systematic selection of implementation strategies, the evidence base behind each strategy was not considered. To mitigate the impact of this potential limitation and to support the utility of the ERIC strategies, we will employ a systematic process of contextualising them using findings from our previous studies and through stakeholder consultation. Finally, whilst we will employ PPI to contextualise the implementation strategies, this study will not include PLWD as study participants although they may be able to provide valuable perspectives based on lived experiences of dementia. Future studies with more time and resources may consider adapting this study [68] to involve PLWD in consensus studies to refine or build on strategies that were identified in the current study.

## Discussion

This study will address the critical gaps in knowledge on how pet robots can be translated from research to clinical practice. This work will carefully consider and integrate multiple, rich sources of data (qualitative data, synthesised literature, stakeholder input, PPI input and the modified Delphi technique), to identify the most relevant strategies to implement pet robots in clinical practice in care homes and nursing home settings. The process will also involve the practical application of theory by using the ERIC taxonomy of implementation strategies and the relatively new CFIR-ERIC tool, to guide the identification and systematic contextualisation of implementation strategies. We have also carefully considered the potential limitations and made efforts to mitigate these within the time and resource constraints. Findings will have practical utility for academic researchers, clinicians and organisations, as they provide a practical starting point to support the implementation of pet robots in care homes and nursing homes for residents with dementia.

In line with principles of good dissemination [69], findings from this study will be disseminated to all key stakeholder groups through different platforms, including a peer-reviewed publication and national and international conference presentation(s), through social media, websites and newsletters for different audiences.

## Supplementary Information

**Additional file 1.** CREDES checklist.

## Data Availability

Not applicable.

## Abbreviations

CFIR: Consolidated Framework of Implementation Research
ENRICH: Enabling Research in Care Homes
ERIC: Expert Recommendations for Implementing Change
PLWD: People living with dementia
