# Supplementary material for: Strategies for implementing pet robots in care homes and nursing homes for residents with dementia: protocol for a modified Delphi study
Source: Implement Sci Commun. 2022 Jun 3;3:58. doi: 10.1186/s43058-022-00308-z (PMC9164492; doi:10.1186/s43058-022-00308-z)
Supplement: Supplementary file 1 — Additional file 1. CREDES checklist. [file 43058_2022_308_MOESM1_ESM.docx]

**Additional File 1: CREDES checklist**

| **CREDES Items** | **Reported in Page** |
| --- | --- |
| Rationale |  |
| 1. Provide a rationale/justify the choice of using the Delphi technique | 6 |
| Planning and design |  |
| 1. State the aims and purpose of the study, and the plans and processes for conducting the Delphi technique. If any modifications are to be made to technique, justifications should be provided and the method used should be systematic and rigorous | 5-6 |
| 1. An a priori criterion for consensus should be defined. This should include clear and transparent guide on: (i) how to proceed with certain items or topics through the survey rounds, (ii) threshold to terminate the Delphi process, and (iii) procedures to be followed, whether consensus is reached/not reached after one or more iterations | 12-13 |
| Study conduct |  |
| 1. All materials provided to the Delphi experts at the outset of the project should be carefully reviewed and piloted in advance in order to examine the effect on experts’ judgement and prevent bias | Not applicable at this stage |
| 1. Researchers need to take measures to avoid influencing the experts’ judgements. If the researcher has a conflict of interest, it is recommended to seek an independent researcher to coordinate the Delphi responses | Not applicable at this stage |
| 1. Interpretation of results: Consensus may not necessarily just refer to the right answer or judgement. Non consensus and stable disagreement provide important insights and highlight differences in perspectives | Not applicable at this stage |
| 1. The final draft of the resulting best practice guidelines should be reviewed and approved by an external board or authority (for external validation) before publication and dissemination | Not applicable at this stage |
| Reporting |  |
| 1. The purpose of the study should be clearly defined, and the appropriateness of using the Delphi technique to address the research objectives must be rationalised | 5-6 |
| 1. Information about the expert panel should be clearly described - Criteria for the selection of experts and information on the recruitment process, experts’ sociodemographic details (including their expertise regarding the research topic), response and non-response rates over the Delphi rounds should be reported | 8-11 |
| 1. The method should be clearly described – This should include information about preparatory steps (i.e., how was available evidence on this topic synthesised), piloting of material and survey instruments, design of the survey, the number and design of survey rounds, data analysis methods, how responses are proceeded in preparation for next Delphi rounds, and methodological decisions taken by the researcher | 6-8, |
| 1. The procedure of the Delphi process (including the preparatory phase, actual Delphi rounds, interim steps of data processing and analysis, and concluding steps) should be illustrated using a flow chart | 7 |
| 1. The definition and attainment of consensus should be clearly stated, so that the reader can comprehend how consensus was achieved, and how non-consensus is dealt with | 11 |
| 1. It is highly advisable to report results separately for each round, so that the evolving consensus over the different rounds can be made transparent. This may include figures to show average group responses, changes between rounds, as well as any modifications to the survey e.g., deletion, addition or modification of survey items based on previous rounds) | Not applicable at this stage |
| 1. The researcher should critically reflect and report on potential limitations and their impact on the resulting guidance | 14 |
| 1. The conclusions should adequately reflect the outcomes of the Delphi study in relation to the scope and applicability of the resulting practice guidelines | 15 |
| 1. The resulting guidance should be clearly identifiable from the publication, including recommendations for translation into practice and implementation.  If the publication does not allow for a detailed presentation of either practice guidance or the methodological features of the applied Delphi technique (or both), reference to a more detailed presentation elsewhere should be made (e.g. availability of the full guideline, publication of a separate paper reporting on methodological details and particularities of the process) | Not applicable at this stage |
